# A comprehensive insight into effects of green tea extract in polycystic ovary syndrome: a systematic review

**DOI:** 10.1186/s12958-021-00831-z

**Published:** 2021-09-23

**Authors:** Vahid Maleki, Ehsaneh Taheri, Parisa Varshosaz, Fatemeh Pourteymour Fard Tabrizi, Jalal Moludi, Hamed Jafari-Vayghan, Mahdi Shadnoush, Seyed Hossein Yahyazadeh Jabbari, Mehri Seifoleslami, Mohammad Alizadeh

**Affiliations:** 1 Student Research Committee, Tabriz University of Medical Sciences, Tabriz, Iran; 2 Clinical Cancer Research Center, Milad General Hospital, Tehran, Iran; 3 Gynecology Department, Khanevade Hospital, AJA University of medical science, Tehran, Iran; 4 Department of Clinical Nutrition, Faculty of Nutrition and Food Sciences, Tabriz University of Medical Sciences, Tabriz, Iran; 5 Student Research Committee, Shahid Beheshti University of Medical Sciences, Tehran, Iran; 6 Departments of Chemistry and Biochemistry, and Biology and Biomolecular Sciences Program, Laurentian University, Sudbury, ON Canada; 7 Nutrition Research Center, Faculty of Nutrition and Food Sciences, Tabriz University of Medical Sciences, Tabriz, Iran; 8 Department of Nutrition, Faculty of Nutrition Sciences and Food Technology, Kermanshah University of Medical Sciences, Kermanshah, Iran; 9 School of Health, Arak University of Medical Sciences, Arak, Iran; 10 Department of Clinical Nutrition, Faculty of Nutrition & Food Technology, Shahid Beheshti University of Medical Sciences, Tehran, Iran

**Keywords:** Green tea extract, Polycystic ovary syndrome, Insulin resistance, Testosterone

## Abstract

**Background:**

Polycystic ovary syndrome (PCOS), as the most common endocrine disorder in reproductive-aged women, is characterized by oxidative stress and ovarian tissue inflammation. Green tea extract (GTE) potentially possesses therapeutic effects for PCOS because of the antioxidant and anti-inflammatory compounds. This systematic review evaluates the potential roles of GTE on metabolic variables, hormone levels, and ovarian function in PCOS.

**Methods:**

A systematic review was conducted of published studies reporting the effects of GTE on PCOS. Several major databases, including PubMed, SCOPUS, and Google Scholar, were searched up from inception to April 2021. Clinical trials and animal studies that assessed the effects of GTE on PCOS were eligible for inclusion.

**Results:**

Of 314 articles found in the search, four human studies and four animal studies were included. All studies in humans showed the effects of GTE on weight loss. GTE’s effect on decreasing testosterone levels in humans and LH levels in animals were also reported. In addition, increases in FSH and progesterone levels in animal models were observed. Although GTE improved fasting blood sugar and insulin levels, the effect of GTE on inflammatory parameters, such as TNF-alpha and IL-6 and antioxidant status, was limited to animal studies.

**Conclusion:**

Therefore, this review suggests that GTE could be considered a potential agent to attenuate PCOS complications mainly due to its effect on weight loss and glycemic levels. However, more studies are needed to formulate conclusions about the effects and mechanisms of GTE in PCOS.

## Background

Polycystic ovarian syndrome (PCOS) affects about 5-20% of women of reproductive ages [1] and is one of the most common and complicated hormonal disorders. This condition is accompanying by clinical symptoms, including irregular menstruation, increased androgens, hirsutism, severe acne, obesity, insulin resistance, Type 2 diabetes, cardiovascular disease (CVD), non-ovulation, and infertility [2]. Although the exact etiology of PCOS is not entirely understood, it seems that genetic factors and environmental play essential roles in the development of PCOS [3]. In general, four sing have been applied for the occurrence of PCOS: (a) an abnormal morphology of the ovaries [4], (b) an overproduction of androgens from ovaries [5], (c) a functional defect in the hypothalamic-pituitary axis and an increase in the secretion of LH/FSH [6], and (d) insulin resistance and hyperinsulinemia [7]. On the whole, hyperandrogenism, insulin resistance, and hyperinsulinemia are underly of metabolic changes and clinical signs in pathogenesis patients with PCOS [8]. Complementary therapies and alternative medicine are common in patients with PCOS to prevent, control, and reduce its complications [9].

Green tea is a widely popular beverage worldwide, and its healthful properties have been extensively explored [10]. Green tea is produced by drying *Camellia sinensis* leaves [11]. The most well-known feature of this beverage is its antioxidant activities [12], which result from high concentrations of polyphenolic compounds, particularly catechins [13]. The biological effects of green tea are mainly attributed to the tea polyphenols, including epigallocatechin-3-gallate (EGCG), epigallocatechin (EGC), epicatechin-3-gallate (ECG), and epicatechin (EC). Of these, EGCG is the most abundant and biologically active constituent [14]. Studies have shown that green tea extract (GTE), as a strong antioxidant compound, has anti-obesity [15], anti-diabetes [16], anti-cardiovascular [17], anti-angiogenesis [18], anticancer [19] and neuroprotective properties [20].

Despite many studies that examined the potential effects of green tea extract (GTE) on metabolic variables, hormone levels, and ovarian function in PCOS, there is no systematic review that summarizes the results of current studies. Hence, the aim of this systematic review is to examine current knowledge about GTE and determine gaps and future directions of GTE in PCOS.

## Methods

### Search strategy

A literature search was conducted in PubMed, SCOPUS, Embase, ProQuest and Google Scholar electronic databases using the keywords “green tea extract” OR “epigallocatechin gallate” OR “*Camellia sinensis*” OR “Catechins” OR “EGCG” OR “polyphenol” OR “epigallocatechin-3-gallate” OR “Epicatechin gallate” OR “Epigallocatechin” OR “flavan-3-ols” AND “polycystic ovary syndrome” OR “PCOS” OR “sclerotic ovary syndrome” OR “dysmetabolic Syndrome”. Reference lists and related records were manually reviewed. The search was limited to English language articles and articles with English abstracts published up from inception to April 2021. The Preferred Reporting for Systematic Reviews (PRISMA) framework was used to conduct this systematic review.

### Eligibility criteria

Inclusion criteria for eligibility included all clinical trials and animal studies that evaluate the potential roles of green tea extract (GTE) on polycystic ovary syndrome. Also, all in vitro models, letters, comments, short communication, abstracts, and those conducted with pregnant/post-partum animals and pregnant or lactating women were excluded.

### Data extraction

Titles and abstracts of the studies were assessed by two independent reviewers, based on the inclusion and exclusion criteria, and the full text of those meeting the inclusion criteria was read in detail to help make the final judgment on their eligibility. Evaluation of the articles was completed according to a checklist of aims, research questions, and inclusion and exclusion criteria. After that, a third reviewer assessed the quality of the selected studies. Two reviewers extracted the data of the eligible full-text articles in detail. Any disagreement between the reviewers during the processing of papers was resolved by discussion among all the reviewers.

## Results

### Selected articles

**Fig. 1:**
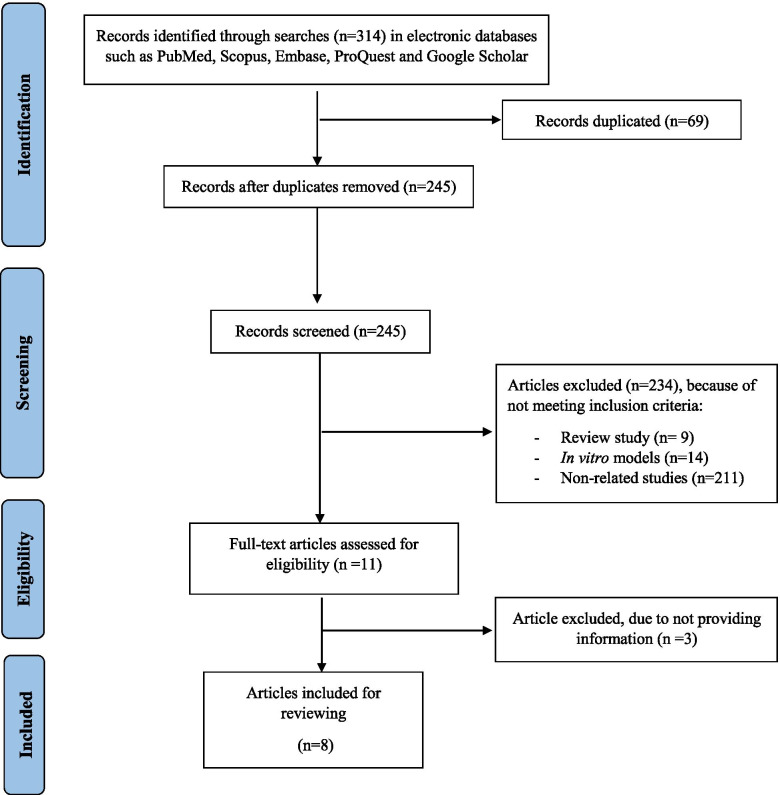
Flow diagram of the literature search and study selection process

A flowchart of the selection process is presented in Fig. 1. In total, after a search of electronic databases, 314 studies were retrieved, 69 duplicate studies were removed, and 245 studies were screened. Of these, 234 studies did not meet the inclusion criteria, and 11 studies were excluded due to the format (letter or comment). Finally, eight studies were included in this systematic review. Characteristics and study outcomes of the eight included articles in the systematic review are summarized in Table 1.

**Table 1:** Characteristics of studies that reported the potential roles of green tea extract (GTE) on PCOS

Type of study	Authors/date	Model	Daily dose	Duration	Results
Animal studies	Sadoughi et al. 2017 [21]	42 Wistar female rats were divided into 7 equal groups: control, PCOS control, PCOS treated with aqueous extract of green tea (50 and 100 mg/kg, PCOS treated with catechin (50 and 100 mg/kg) and PCOS treated with clomiphene citrate (1.5 mg/kg, 24 days).	50,100 mg/kg/day GTE	24 day	- Serum level of LH, β-estradiol, and testosterone significantly decreased. - Serum level of FSH, progesterone significantly increased. - Number of preantral, antral, preovulatory follicles, and corpus luteum significantly increased.
	Rahbarian et al. 2017 [22]	24 Wistar female rats were divided into 4 equal groups: control (saline solution), non-treated PCOS (saline solution) and PCOS treated with catechin (50 and 100 mg/kg, 24 days).	50,100 mg/kg/day GTE	24 day	-Serum levels of TNF-α, IL-1β, MDA and IL-6 decreased -SOD, CAT, GPX in ovarian tissue significantly increased.
	Ghafurniya et al. 2015 [23]	96 Wistar rats were divided into 4 groups: PCOS group, three experimental groups, which were given 50, 100 and 200 mg/Kg BW green tea extract 10 days, intraperitoneally.	50,100,200 mg/kg/day GTE	10 day	-LH serum level, body weight and ovarian weight decreased -Insulin resistance and blood glucose decreased -Improved of ovarian function
	Ghafurniya et al. 2014 [24]	40 Wistar rats were divided into 5 groups: PCOS rats received no injections (PCOS control), rats received saline (sham), PCOS rats that received of 50 mg/kg of green tea extract (GT-Treated-1), rats that were received intraperitoneal injection 100 mg/kg of green tea extract (GT-Treated-2), rats that were received intraperitoneal injection 200 mg/kg of green tea extract (GT-Treated-3).	50,100,200 mg/kg/day GTE	2 weeks	–CRP and IL-6 serum levels reduced. –Improved follicular layers thickness (improving of ovarian function)
Human studies	Mombaini et al. 2017 [25]	45 women with PCOS were randomly allocated into 2 groups receiving GT tablets or placebo	500-mg/day GTE	1.5 months	- Significant decrease in BMI, body weight, waist circumference, and body fat percentage. -No effect on the levels of inflammatory factors (IL-6, hs-CRP, TNF-α)
	Tehrani et al. 2017 [26]	60 Overweight women with PCOS were given GTE	500 mg/day GTE capsules	3 months	– Significant decrease in Body weight, FBG, serum insulin and free testosterone.
	Allahdadian et al. 2015 [27]	60 overweight or obesity women with PCOS were received GTE tablets and placebo.	500 mg/day GTE	3 months	- Significant decrease in BMI and body weight. -Significant decrease in FBS, in sulinand free testosterone levels
	Chan et al. 2005 [28]	34 obese Chinese women with PCOS were randomized into either treatment with GTE capsules or placebo.	1.5 cups of 2% Lung Chen tea (an equivalent of 540 mg EGCG)	3 months	-No significant effect on body weight, BMI, body fat, total testosterone, SHBG, Free androgen index, DHEA-S, FSH, LH.

*Abbreviations*: *BMI* Body mass index, *CAT* Catalase, *DHEA* Dehydroepiandrosterone, *FPG* Fasting plasma glucose, *GTE* Green tea extract, *LH* Luteinizing hormone, *FSH* Follicle-stimulating hormone, *hs-CRP* High sensitivity C-reactive protein, *GPX* Glutathione peroxidase, *MDA* Malondialdehyde, *LDL* Low-density lipoprotein, *HDL* High-density lipoprotein, *IR* Insulin resistance, *IL-6* Interleukin 6, *SOD Superoxide dismutase*, *TNF-α* Tumor necrosis factor

### The effects of green tea extract (GTE) on weight loss in PCOS

Allahdadian et al. [27] demonstrated statistically significant weight loss in overweight and obese PCOS women after prescribing two capsules of GTE 500 mg/day for 12 weeks (*P* < 0.05) [27]. Similarly, Mombaini et al. reported that body weight, BMI, waist circumstance (WC), and body fat percentage decreased statistically significantly after GTE 500 mg/day for 45 days in women with PCOS (*P* < 0.05) [25]. Moreover, a study by Tehrani et al. found that 500 mg GTE supplementation for 12 weeks led to a statistically significant weight reduction among patients with PCOS (*P* < 0.05) [26]. In contrast, Chan et al. [28] reported no statistically significant effects of consuming 540 mg of EGCG for 3 months on weight reduction in obese PCOS women compared to a control group (*P* > 0.05) [28]. Also, in an animal study, Ghafurniyan et al. reported a statistically significant weight decrease in PCOS model rats after administration of GTE for 10 days (*P* < 0.05) [23].

### The effects of green tea extract (GTE) on glycemic status in PCOS

Insulin resistance and hyperglycemia are the most common features of patients with PCOS [29]. In this regard, Ghafurniyan et al. [24] reported positive effects of statistically significant from a 200 mg/kg injection of GTE for 10 days on insulin level in PCOS model rats [24]. Similarly, a statistically significant decrease in insulin resistance was reported after consumption of GTE in obese and overweight PCOS women (*P* < 0.05) [26]. Allahdadian et al. [27] found that GTE supplementation (500 mg/day) for 3 months led to a statistically significant decrease in serum insulin and FBS levels (*P* < 0.05). Likewise, Tehrani et al. [26] showed that 500 mg of GTE supplementation for 3 months in overweight women with PCOS decreased serum levels of FBS, insulin, and free testosterone (*P* < 0.05). However, Chan et al. [28] found that administration of 1.5 cups of Lung Chen tea, as an equivalent of 540 mg EGCG, for 3 months in obese women with PCOS did not yield any effect on FBS and fasting insulin.

### The effects of green tea extract (GTE) on hormone state and ovarian function in PCOS

In a study by Ghafurniya et al., a statistically significant decreasing effect of GTE injection on theca cell layer thickness and number of follicular cysts were seen in PCOS model rats [24]. In another animal study, Sadoughi et al. found that GTE administration over 24 days led to a statistically significant decrease in serum levels of LH, β-estradiol, and testosterone in PCOS model rats, as well as improved ovarian function (Significant increase in the number of preantral, antral, prehavulatory and corpus luteum follicles and also caused a significant decrease in the number of cystic follicles), FSH, and progesterone levels, were also reported [21].

Allahdadian et al. [27] reported that administration of GTE (500 mg/day) for 12 weeks had a beneficial effect on reproductive outcomes, especially in decreasing serum-free testosterone levels in obese subjects with PCOS. Additionally, Tehrani et al. found that patients who received GTE (500 mg/day) had significantly decreased free testosterone levels compared with placebo subjects [26]. However, Chan et al. [28] showed that administration of 1.5 cups of Lung Chen tea for 3 months did not lead to significant changes in hormonal profiles, including testosterone, SHBG, free androgen index, DHEA-S, FSH, and LH.

### The effects of green tea extract (GTE) on inflammation and oxidative stress in PCOS

Ghafurniyan et al. found statistically significant reducing effects of GTE by injection on IL-6 and CRP biomarkers after 14 days’ treatment in PCOS model rats (*P* < 0.05) [24]. Likewise, Mombaini et al. [25], in a clinical trial conducted in PCOS patients, found that 500-mg/day of GTE supplementation for over 6 weeks did not lead to statistically significant changes in levels of inflammatory factors (IL-6, hs-CRP, TNF-α). However, Rahbarian et al. [22] reported that ingestion of GTE for 24 days in PCOS model rats statistically significantly increased serum levels of TNF-α, IL-1β, MDA, and IL-6 and increased SOD, CAT, and GPX in ovarian tissue (*P* < 0.05).

## Discussion

Weight gain is one of the clinical features of patients with PCOS who are both genetically predisposed and exposed to environmental risk factors [29]. Besides the effect of genetic factors in PCOS etiology, changes in lifestyle, particularly weight loss, are important to improve infertility and PCOS- related metabolic disorders in women with PCOS [30].

Clinical trials conducted in women with PCOS have found significant positive effects of GTE on weight loss. In four studies, three reported that 500 mg/day extract of GTE caused a decrease in weight in women with PCOS. In all four studies, the dosage of green tea extract was 500 mg per day, but the duration of the interventions ranged from 6 weeks to 3 months [25–27]. However, Chan et al. found no significant effect of GTE was reported on body weight, fat percentage, and BMI in obese women with PCOS. The inconsistency between the results of Chan et al. and the other studies with significant effects of GTE on weight loss may be explained by the difference in the type of GTE and the process of tea extraction: in Chan et al., PCOS women consumed 1.5 cups of 2% Lung Chen tea, which was equal to 540 mg EGCG [28]. In addition to weight loss, Mombaini et al. demonstrated the effect of GTE on waist circumference and body fat percentage [25].

Regarding the mechanism, GTE is a thermogenic agent that reduces body weight by increasing energy expenditure and fat oxidation [25, 31, 32]. This feature can be described by caffeine and catechin polyphenols in GTE, which inhibits catechol-0-methyltransferase (COMT) as norepinephrine degrading enzymes, leading to increased levels of norepinephrine and prolonged action of catecholamines. Decreased appetite and a reduction in the absorption of nutrients such as dietary lipids and glucose are other mechanisms that contribute to the weight loss effect of GTE [33–36].

Green tea extract (GTE) has also exhibited the potential to improve glycemic parameters in women with PCOS. There is convincing evidence that ameliorating insulin resistance and hence reducing circulating insulin levels leads to the improvement of hormonal imbalance and resumption of ovulation in PCOS subjects [37]. In two studies conducted separately by Tehran et al. [26] and Allahdadian et al. [27], supplementation of GTE supplementation at 500 mg/day showed significant reductions in the FBS and insulin levels. In both studies, GTE supplementation was administrated for 3 months for overweight or obese women with PCOS. In another experimental study, the same results were observed in PCOS model rats after administration of GTE for 10 days [23]. Studies suggest that GTE increases basal and insulin-stimulated glucose uptake from adipocytes and also inhibits glucose absorption in the small intestine [10, 38].

Furthermore, GTE studies have demonstrated that insulin resistance (IR) is ameliorated by augmented expression of glucose transporter 4 (GLUT-4) in adipocytes [39]. In addition, GTE’s antidiabetic activity suppresses gluconeogenesis in hepatic cells [40]. There is evidence suggesting that GTE has insulin-like and insulin-enhancing activities [41, 42]. Based on the results of our review in the effects of GTE on decreasing FBS and insulin levels, we expected that insulin resistance would also be reduced by GTE; however, HOMA-IR, as an index of insulin resistance which is calculated according to FBS and insulin, was not evaluated in any of the included studies.

PCOS is a metabolic disorder along with reproductive hormonal abnormality, including over-production of androgenic hormones such as testosterone, androstenedione, and progesterone, as well as increased secretion of LH to FSH [43]. In addition, insulin resistance is also more prevalent in patients with PCOS that leads to higher androgen levels and an increased LH/FSH ratio [44]. GTE may reduce the adverse effects of hormonal disturbances in polycystic ovaries by regulating the secretion of gonadotropins or its receptors by regulating GnRH pulsatility [22, 28]. Findings from our review showed that GTE supplementation could lead to a reducing effect on serum levels of LH, free testosterone, and β-estradiol, but the serum levels of FSH and progesterone significantly increased in PCOS [22, 23, 26, 27]. After administration of GTE the increase in progesterone levels causes a reduction in LH with a feedback effect on the pituitary-ovarian axis and an increase in ovulation rates in polycystic ovaries [21, 28, 45]. In one animal study, GTE showed more favorable effects on improving hormonal parameters in rats with PCOS. In addition, GTE flavonoids may inhibit the activity of the aromatase enzyme, which has a major role in the synthesis of estradiol in ovarian granulosa cells [21]. However, most studies regarding the effects of GTE on hormonal status in PCOS were obtained from animal studies; definite conclusions regarding GTE in improving the hormonal profile of PCOS women will need more studies in humans with different dosages of GTE.

A hyperandrogenism state in PCOS can induce inflammation and promote oxidative stress through insulin resistance and, conversely, stimulate inflammation with insulin resistance that may enhance excess production of ovarian androgen [46, 47].

In rat models of PCOS, administration of GTE increased the activity of antioxidant enzymes of SOD, CAT, and GPX in the ovarian tissue, decrease inflammatory cytokines, including Il-6, TNF-α, IL-1β, CRP and DNA oxidative damage, and serum MDA levels [22, 24].

According to our knowledge, only one randomized control trial investigated the effect of 500 mg/day of GTE supplementation for 6 weeks on inflammation biomarkers among women with PCOS and reported no significant change in levels of IL-6, TNF-α, and hs-CRP [25]. The findings of our review confirm the results of two systematic reviews and a meta-analysis of randomized clinical trials that showed that GTE supplementation had no effect on inflammatory biomarkers [48, 49]. There appear to be differences between studies due to the baseline status of inflammation and the oxidation status of participants, the type, dosage, and duration of GTE extraction. In addition, adjusting confounders in studies makes it difficult to have a consistent conclusion regarding the anti-inflammatory effects of GTE.

**Fig. 2:**
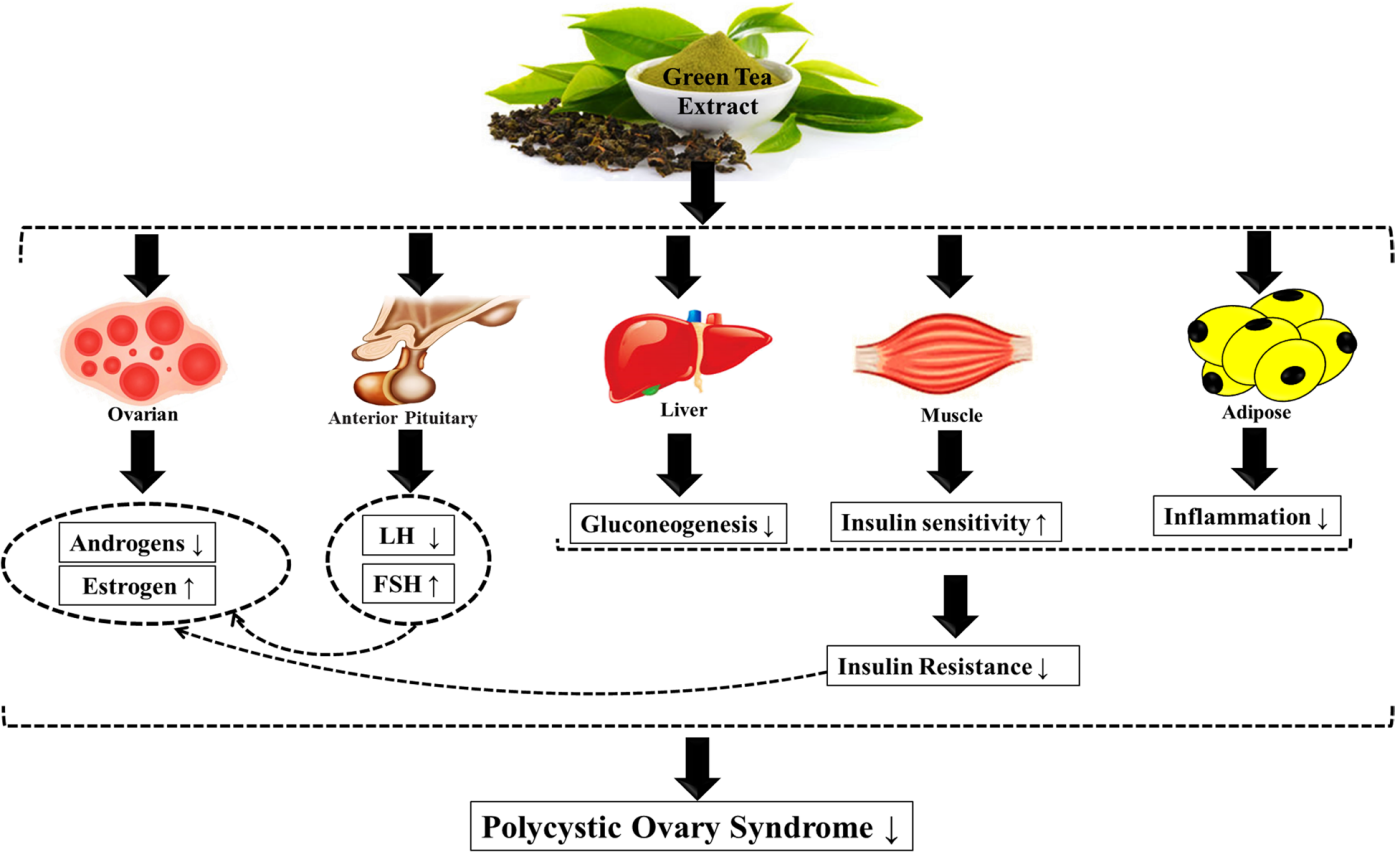
Impacts of green tea extract (GTE) on metabolic variables in polycystic ovary syndrome

As a whole, the potential roles of the effect of green tea extract (GTE) in polycystic ovary syndrome are summarized in Fig. 2.

### Knowledge gaps and future directions

This is the first systematic review about the potential of green tea extract (GTE) in the management of PCOS. Several gaps were identified for consideration in future studies. None of the included studies assessed the effect of GTE on clinical symptoms of PCOS, such as hirsutism and acne. Different GTE preparations alter the availability of bioactive compounds of GTE and should be considered along with the dosage. Furthermore, catechins, as the most important polyphenols of GTE, are needed to be studied for their contribution to the anti-androgenic effects of GTE. Therefore, independent tests of the composition of GTE should be conducted to recognize effective compounds for PCOS patients. Future clinical trials with longer duration and higher dosage should aim to categorize the exact effects of GTE metabolic variables in patients with PCOS.

## Conclusion

Current evidence indicates that green tea extract (GTE) supplementation has potential beneficial effects on PCOS. Despite a lack of human studies on ovarian histology, animal studies support the impact of GTE in improving ovarian function and histology. Moreover, GTE could lead to improving glycemic control in PCOS and may decrease body weight, LH, and androgens in PCOS patients. However, conclusions about the effect of GTE on inflammation were contradictory. There are not currently enough RCTs/definitive studies to make robust conclusions about the effect of GTE on oxidative stress in PCOS.

## Data Availability

All data generated or analyzed are included in the results of the manuscript.

## Abbreviations

BMI: Body mass index
CAT: Catalase
DHEA: Dehydroepiandrosterone
FPG: Fasting plasma glucose
FSH: Follicle-stimulating hormone
GPX: Glutathione peroxidase
GT: Green tea
HDL: High-density lipoprotein
hs-CRP: High sensitivity C-reactive protein
IL-6: Interleukin 6
IR: Insulin resistance
LDL: Low-density lipoprotein
LH: Luteinizing hormone
MDA: Malondialdehyde
SOD: Superoxide dismutase
TNF-α: Tumor necrosis factor
